# UV-C side-emitting optical fiber-based disinfection: a promising approach for infection control in tight channels

**DOI:** 10.1128/spectrum.00040-24

**Published:** 2024-04-30

**Authors:** Muhammad Salman Mohsin, Melisa Avdic, Katrina Fitzpatrick, Mariana Lanzarini-Lopes

**Affiliations:** 1 Environmental and Water Resources Engineering, Department of Civil and Environmental Engineering, University of Massachusetts Amherst, Amherst, Massachusetts, USA; NHLS Tygerberg/Stellenbosch University, Cape Town, Western Cape, South Africa

**Keywords:** endoscope, UV-C LED, *E. coli*, *P. aeruginosa*, *MRSA*, PTFE channel

## Abstract

The growth of pathogenic bacteria in moist and wet surfaces and tubing of medically relevant devices results in serious infections in immunocompromised patients. In this study, we investigated and demonstrated the successful implementation of a UV-C side-emitting optical fiber in disinfecting medically relevant pathogenic bacteria (*Pseudomonas aeruginosa* and Methicillin-resistant *Staphylococcus aureus* [MRSA]) within tight channels of polytetrafluoroethylene (PTFE). PTFE is a commonly used material both in point-of-use (POU) water treatment technologies and medical devices (dental unit water line [DUWL], endoscope). For a 1-m-long PTFE channel, up to ≥6 log inactivation was achieved using a 1-m-long UV side-emitting optical fiber (SEOF) with continuous 16-h exposure of low UV-C radiation ranging from ~0.23 to ~29.30 μW/cm^2^. Furthermore, a linear model was used to calculate the inhibition zone constant (k`), which enables us to establish a correlation between UV dosage and the extent of inactivated surface area (cm^2^) for surface-bound *Escherichia coli* on a nutrient-rich medium. The k` value for an irradiance ranging from ~150 to ~271.50 μW/cm^2^ was calculated to be 0.564 ± 0.6 cm·cm^2^/mJ. This study demonstrated the efficacy of SEOFs for disinfection of medically relevant microorganisms present in medically and domestically relevant tight channels. The impact of the results in this study extends to the optimization of operational efficiency in pre-existing UV surface disinfection setups that currently operate at UV dosages exceeding the optimal levels.

**IMPORTANCE:**

Germicidal UV radiation has gained global recognition for its effectiveness in water and surface disinfection. Recently, various works have illustrated the benefit of using UV-C side-emitting optical fibers (SEOFs) for the disinfection of tight polytetrafluoroethylene (PTFE) channels. This study now demonstrates its impact for disinfection of medically relevant organisms and introduces critical design calculations needed for its implementation. The flexible geometry and controlled emission of light in these UV-SEOFs make them ideal for light distribution in tight channels. Moreover, the results presented in this manuscript provide a novel framework that can be employed in various applications, addressing microbial contamination and the disinfection of tight channels.

## INTRODUCTION

The growth of pathogenic bacteria on wetted surfaces such as tight channels used in point-of-use (POU) plumbing, medical equipment (endoscopes, dental unit water line [DUWL], etc.) is a concerning issue and can lead to fouling and various infection problems in hospitals (1), food processing plants (2), and water distribution lines, etc. (3, 4). One such well-known example of contamination and infection in tight channels is the use of reprocessed gastrointestinal (GI) endoscopes in the healthcare industry. These expensive and indispensable heat-sensitive medical equipment must be disinfected using high-level disinfectants (HLDs) in between use to prevent transmission of infection from one patient to another. The common reprocessing protocol for a GI endoscope includes seven steps: precleaning, cleaning, rinsing, disinfection with a high-level disinfectant, rinsing, drying, and storage (5). Despite all these precautionary measures, there continue to be outbreaks of endoscope-related infections (6). In the USA, over 17.7 million cases of GI endoscopic procedures are performed annually. The infection rate following a GI endoscopic procedure was calculated to be 0.2%, which is 35,400 patients per year (7).

The most common microorganisms associated with infections were reported to be *Pseudomonas aeruginosa (P. aeruginosa*) followed by other *Enterobacteriaceae* groups (*Escherichia coli*, *Enterobacter*, etc.) and gram-positive cocci (Methicillin-resistant *Staphylococcus aureus* [MRSA], etc.) (7). In 2017, 32,600 infections among hospitalized patients and 2,700 deaths were estimated to be caused by multidrug-resistant *P. aeruginosa*. An additional 120,000 bloodstream infections and 20,000 deaths caused by MRSA were reported in the USA (8, 9). Approximately, 10 billion US$ is spent every year to treat MRSA, averaging ~US $60,000–70,000 per patient in the USA (10). In this study, our work is focused on major reported infection-causing pathogenic bacteria (*P. aeruginosa* and MRSA) in medical equipment with tight channels like endoscopes; spores are also problematic in hospital environments and more resistant to HLD than other bacteria and viruses. However, they are also likely to be killed when endoscopes undergo manual cleaning (11).

Moreover, numerous studies have highlighted the development of bacterial biofilms within the narrow channels of DUWL (12, 13). These biofilms pose a potential risk of infection for patients, as they can harbor pathogenic bacteria, including *P. aeruginosa*, MRSA, and *E. coli* (14). Although there are currently no standardized procedures and guidelines for preventing DUWL contamination, manufacturers typically offer recommendations for the use of disinfectants to mitigate the risk of infection. Despite the application of HLDs, there have been reports of biofilm formation and persistence (15).

Cold sterilants such as glutaraldehyde and hydrogen peroxide solutions are commonly used for the sterilization of surgical instruments that cannot withstand thermal or steam disinfection (autoclave). Some cold sterilants can achieve sterilization or high-level disinfection. However, there are reported bacterial contamination with the use of cold sterilization techniques that can be due to inappropriate dilution, incorrect pH during time of application, contamination from particulate and organic matter that are introduced during the process (16). These issues highlight a need of additional disinfection techniques (such as UV) to support reprocessing. Additionally, other concerns of using cold sterilant include lengthy procedure time, contamination through the introduction of particulate or organic matter, and use and disposal of consumables, which can cause environmental concerns (17). A novel approach, such as the distribution of UV radiation into these spaces can mitigate and support many of the existing concerns while ensuring proper disinfection.

Ultraviolet radiation is considered a sustainable approach for disinfection that does not rely on the use of consumables and is an effective disinfection solution for both open surfaces and tight spaces (18, 19). UV-C radiation between the wavelengths of 240 to 280 nm exerts a germicidal impact by inactivating the microorganism through damage to their DNA and protein structures (20–22). Mercury lamps are commonly used for UV disinfection, but their geometric restrictions make them unsuitable for use in tight channels found in medical equipment, water distribution line, and other food processing units.

UV light-emitting diodes (LED) have emerged as a promising alternative to traditional lamps (23–26). The small size of these LEDs enables creative engineering design and is ideal for POU- disinfection applications (27). Other benefits of UV LEDs include the lack of toxic mercury, specific wavelength emission, longer life span, and enhanced durability (23, 28). Previous studies illustrated the potential for UV LEDs for disinfection in appliances such as shower heads (29). However, they also discuss the challenges in designing devices that can distribute UV light throughout the entirety of the appliance. Specifically, numerous LEDs would have to be inserted to effectively irradiate the full inner surface.

**Fig 1:**
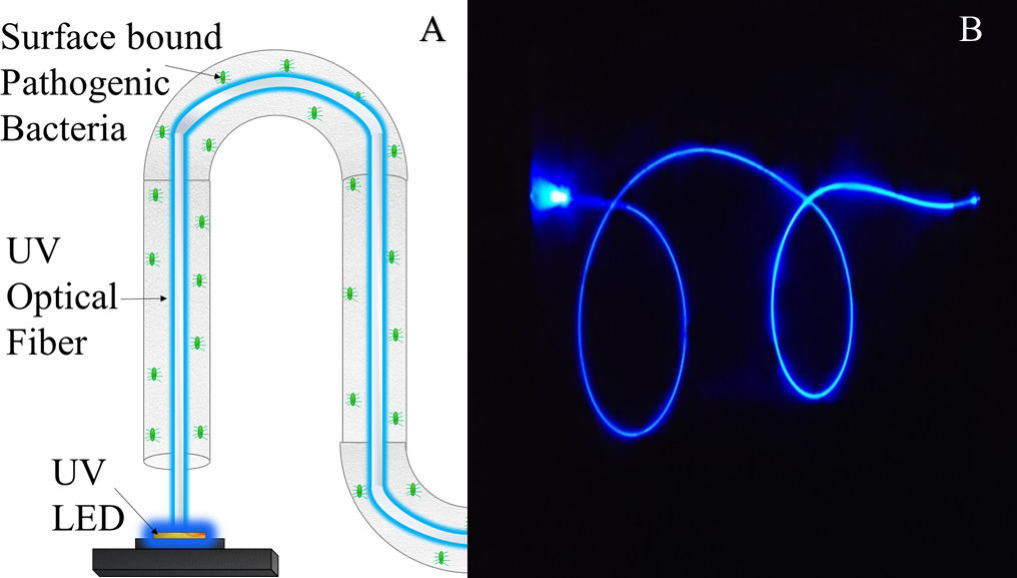
One of the limitations of UV LED disinfection is its geometric restriction, this schematic portrays (**A**) how UV-C optical fiber can get UV light into constricted tight channels and (**B**) the picture of a UV-C SEOF connected to a 265-nm light engine.

Previous studies have proposed an alternative method for light distribution, employing side-emitting optical fibers (UV-C SEOF) to guide and distribute UV-C LED light within confined spaces (19, 30, 31). These publications have illustrated the use of UV-C SEOF for disinfection and biofilm inhibition for open-surface disinfection. Our work now aims to validate the feasibility of using a flexible UV side-emitting optical fiber to disinfect the entire inner surface area of a bent channel for medically relevant microorganisms (see Fig. 1). The primary objective of this investigation is to demonstrate the efficacy of a single UV-C SEOF in deactivating opportunistic pathogens present within narrow channels. The paper also presents a detailed discussion on how engineering parameters such as channel length, diameter, and inhibition zone constant (k’) will influence the effectiveness and time required for disinfection.

## RESULTS

### Calculation of inhibition zone constant of *E. coli* for surface disinfection

**Fig 2:**
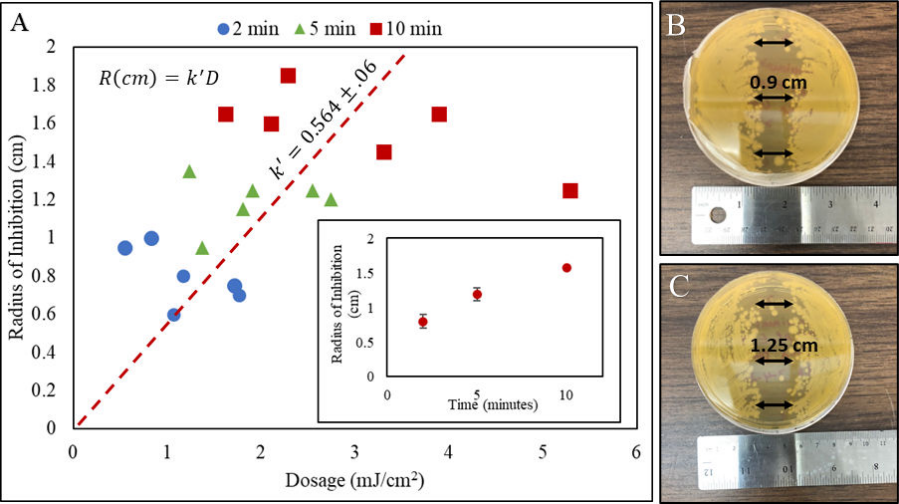
(**A**) UV dose response of *E. coli* with respect to the radius of inhibition. Inset represents the statistically different average radius of inhibition for exposure times of 2, 5, and 10 min. (**B** and **C**) represent the UV-C SEOF irradiated agar plates at exposure times of 2 and 5 min with an average radius of inhibition at 0.9 and 1.25 cm, respectively.

The inhibitory effect of a UV-C optical fiber on an *E. coli* lawn spread on a nutrient-rich media was explored. Figure 2A illustrates the UV dose response of *E. coli* with respect to the radius of inhibition. The dotted line represents the combined linear fit for all three exposure times with an inhibition zone constant of 0.564 ± cm·cm^2^/mJ. The average radius of inhibition for exposure times of 2, 5, and 10 min was measured at 0.8 ± 0.1, 1.2 ± 0.2, and 1.6 ± 0.1 cm, respectively. A linear increase in the radius of inhibition was observed as UV dosage was increased from ~0.7 to ~3.5 mJ/cm^2^. The radius of inhibition did not increase with a further increase in dosage. The UV dosage at the edge of the lawn for maximum radius of inhibition at 10 min varied significantly from ~1.6 to ~5.2 mJ/cm, which supports the previously observed trends that the maximum radius of inhibition does not solely depend on UV dosage (30).

A linear model was used to calculate k' (inhibition zone constant) before achieving the maximum radius. The k' value for an irradiance ranging from ~150 to ~271.50 μW/cm^2^ was calculated to be 0.564 ± 0.6 cm·cm^2^/mJ. The parameter k' enables us to establish a correlation between UV dosage and the extent of inactivated surface area for surface-bound *E. coli* on a nutrient-rich medium.

### UV-C SEOF dose ranges within a channel

**Fig 3:**
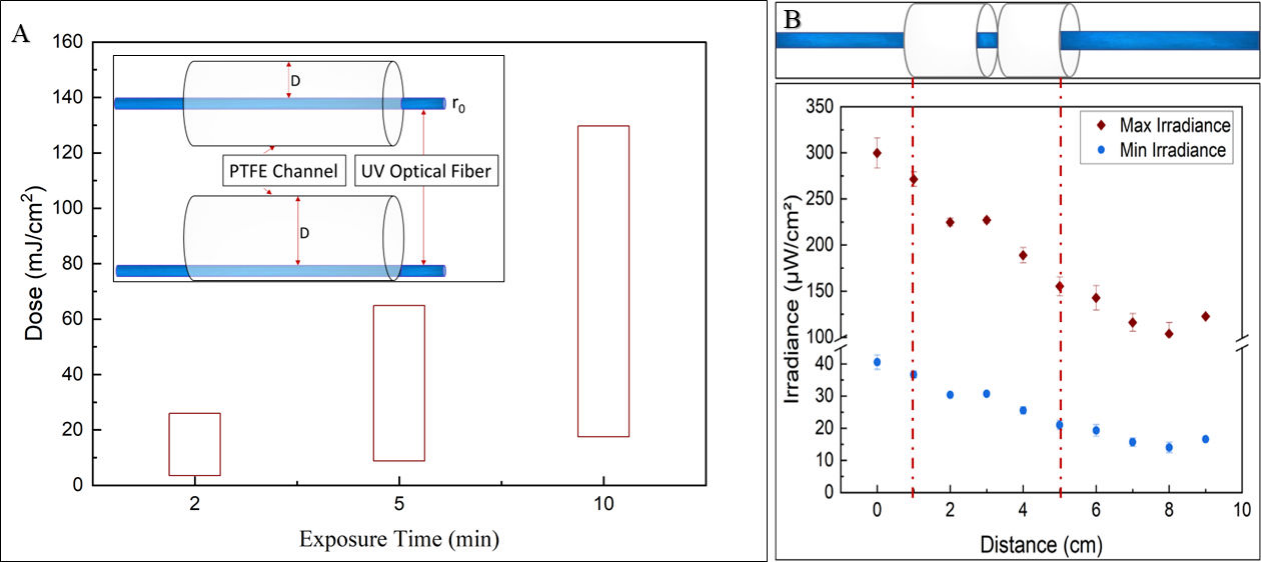
(**A**) Range of UV dosages inside the PTFE channel for exposure times of 2, 5, and 10 min. (**B**) The position of the PTFE channel (2 cm) on UV-C SEOF and the dotted line shows the range of irradiance the channel is exposed to depending on the distance from the UV-C SEOF.

Exposing a polytetrafluoroethylene (PTFE) channel to UV light via UV-C SEOF results in a range of UV irradiance (μW/cm^2^) on the surface of the channel. This is because we cannot perfectly control the position of the flexible fiber inside the channel. Figure 3A presents the range of UV dosages to which the bacteria inoculum is exposed within the PTFE channel for exposure times of 2, 5, and 10 min. The UV dosage (mJ/cm^2^) exhibits a linear increase proportional to the exposure time. Specifically, the range of UV dose exposure within the channel over a 2-min period was calculated as 2.51 to 32.58 mJ/cm^2^, while the 5-min exposure yielded a range of 6.29 to 81.45 mJ/cm^2^. For a 10-min exposure time, the UV dose expanded to 12.58 to 162.9 mJ/cm^2^. As detailed in the second paragraph of dose response of bacterial colonies in nutrient rich media in methodolgy section , the irradiance decreases with distance from the UV-C SEOF, therefore affecting the UV dosage encountered by the bacteria attached to the channel surface (inset Fig. 3A). The range of UV irradiance the channel was exposed to throughout its length is further illustrated in Fig. 3B.

### Inhibitory effect of UV-C SEOF for a 2-cm PTFE channel

**Fig 4:**
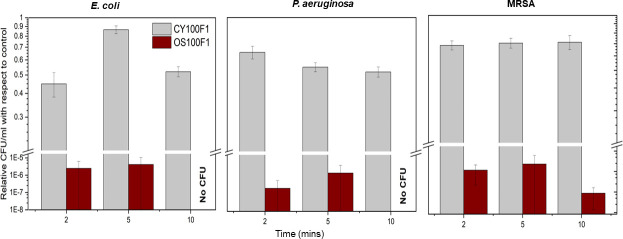
The number of CFUs recovered after UV exposure relative to the control for *E. coli*, *P. aeruginosa*, and MRSA.

A partial PTFE channel (2 cm) filled with a bacterial culture of approximately 10^8^ CFU/mL cell density (*E. coli*, *P. aeruginosa*, and MRSA *separately*) was exposed to a continuous UV-C irradiance range of ~20.97 to ~271.50 μW/cm^2^ as a proof of concept. The minimum irradiance reaching the surface of the channel was calculated by equation (1), where the distance is maximum (3.2 mm) between the UV-C SEOF and the channel (Fig. 3B). The number of colony forming units (CFUs) recovered after UV treatment relative to the no-fiber control for the (i) UV-C SEOF and (ii) Cytop control fiber is re-illustrated in Fig. 4. There was no statistically significant difference (*P* > 0.05, unpaired *t*-test) in inactivation observed for Cytop control fiber for all three bacterial strains. The average number of CFUs recovered after UV exposure relative to the control was 63% (0.20 log reduction). There was a statistically significant distinction of 5.8 log between the number of CFUs recovered after UV exposure with UV-C SEOF compared to the Cytop control fiber. An average of ≥6 log statistically significant reduction with respect to control (*P* < 0.05, two-sample group *t*-test) was achieved after exposing the PTFE channels to UV-C SEOF with exposure times of 2, 5, and 10 min as illustrated in Fig. 4. A break rug was used in the y-axis for visual comparison. There was no statistically significant difference (*P* > 0.05, unpaired *t*-test) in inactivation for the UV-C SEOF for all three bacterial strains.

No CFUs were detected when plated right after UV exposure and were below the detection limit. The cells were allowed to replicate during overnight incubation. The relative CFUs in respect to control in the incubated solution are reported in Fig. 4. At an exposure time of 10 min for both *E. coli* and *P. aeruginosa*, no CFUs were detected even after overnight incubation but that does not indicate that complete sterilization was achieved. As described earlier, there was no statistically significant difference (*P* > 0.05, unpaired *t*-test) in inactivation observed for UV-C optical fiber for all three bacterial strains. The number of CFUs recovered after UV exposure for MRSA for an exposure time of 10 min was slightly higher than *E. coli* and *P. aeruginosa* but statistically not significant to indicate a higher UV resistance of MRSA at the same dosage.

### Inhibitory effect of UV-C SEOF on 1-m PTFE channel

**Fig 5:**
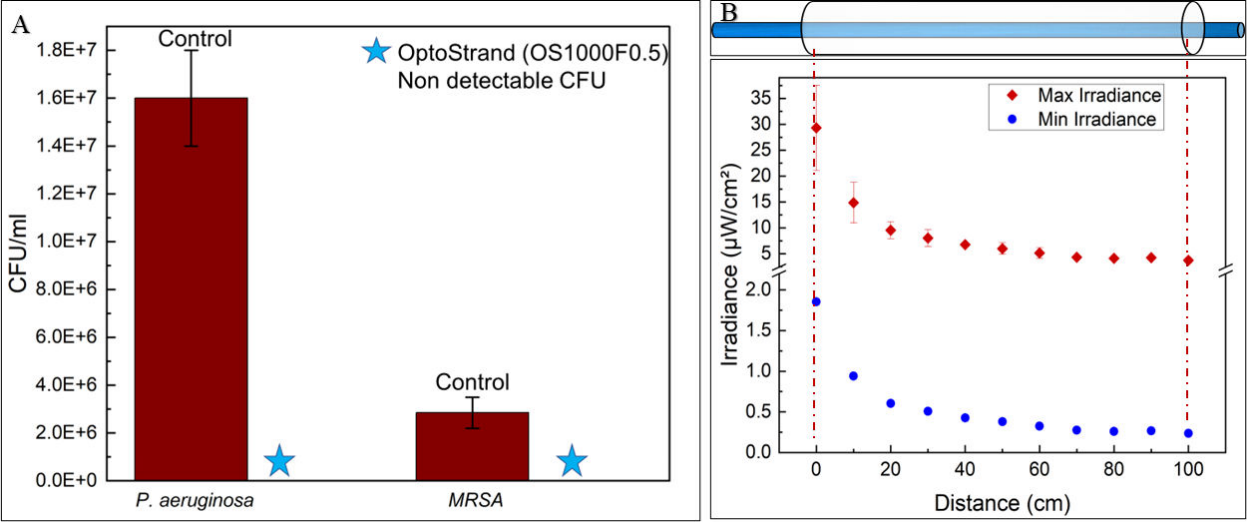
(**A**) The number of CFUs (CFU/mL) recovered after UV exposure relative to the control for *P. aeruginosa* and MRSA. (**B**) The position of the PTFE channel (1 m) on the UV-C SEOF and the dotted line shows the range of irradiance the channel is exposed to depending on the distance from the UV-C SEOF.

A 1-m-long PTFE channel filled with a bacterial culture of approximately 10^7^ and 10^6^ CFU/mL cell density (*P. aeruginosa* and *MRSA separately*) was individually exposed to a continuous low UV-C radiation of ~0.23 to ~29.30 μW/cm^2^ for 16 h. Figure 5B illustrates the exponential decay in UV light emitted from the fiber through the length of the channel. As previously discussed, the inner surface of the fiber will be exposed to a range of UV irradiances due to (i) decreasing light emitted from the fiber and (ii) uncontrolled variation in distance between the fiber and the channel surface. The control for *P. aeruginosa* had higher cell density (10^7^ CFU/mL) as compared to MRSA (10^6^ CFU/mL). These results agree with the bacterial characteristics associated with surface attachment. Specifically, the pili, lipopolysaccharides, exopolysaccharides, and outer membrane proteins of *P. aeruginosa* favor their attachment to biotic and abiotic surfaces (32).

The number of CFUs recovered from the PTFE channel after 16 h of UV exposure relative to the no-UV control is illustrated in Fig. 5A. Similar to the 2-cm channel results, there was an average of ≥6 log reduction in CFU with respect to the control for both bacterial strains. No CFUs were detected when plated right after UV exposure and were below detection limit. The CDC defines sterilization as a process that destroys all forms of microbial life (33). Therefore, by the medical definition, sterilization was not achieved.

## DISCUSSION

The UV dose response of *E. coli* with respect to the radius of inhibition showed that it does not solely depend on UV dosage; it also depends on the susceptibility of the microorganism to UV-C light, the intensity (irradiance) of light (µW/cm^2^) reaching the edge of the inhibition zone, and the type of media used for inoculation (18, 34). Bacterial cell growth at the edge of the inhibition zone can be due to (i) the rate of growth or bacterial DNA repair mechanism exceeding the UV DNA destruction rate (35) or (ii) the irradiance (µW/cm^2^) is insufficient to cause DNA damage to the cell (18). At 1.65-cm distance from the UV-C SEOF, the UV irradiance was ~2.69 µW/cm^2^. This value is different from our previously published work where we calculated the minimum irradiance (µW/cm^2^) required for the complete inactivation of surface-bound *E. coli* to be 0.38 ± 0.11 µW/cm^2^. The variation in values may be attributed to differences in the experimental setups employed in both studies. In the previous study, a lower UV irradiance source of approximately 1.6 µW/cm^2^ was used, with an exposure duration ranging from 18 to 24 h. In contrast, the present experimental configuration involved a higher UV irradiance source of approximately 103.7 to 224.8 µW/cm^2^, with exposure periods of 2 to 10 min. These results suggest that varying levels of inactivation would be observed when employing higher or lower irradiance exposures for the same UV dosage. However, a detailed study is needed to further understand bacterial dose response at a wide range of irradiance for different wavelengths (nm).

The inhibition zone constant (k') calculated in this study serves as a valuable tool for engineering computations in the design of UV surface disinfection systems. Inhibition zone constant allows for the determination of the necessary UV dosage to attain a specific disinfection area, and conversely, to gauge the surface area achievable with a given UV dosage. Its application extends to the optimization of operational efficiency in pre-existing UV surface disinfection setups that currently operate at UV dosages exceeding the optimal levels. Lanzarini-Lopes et al. (30) previously reported a maximum radius of inhibition of ~1.45 cm at a UV dosage of 4.3 mJ/cm^2^ for surface-bound *E. coli* with ridged SEOFs, but the k' value had not been calculated. These results enabled the quantification of the expected inhibition zone around a linear UV-C source for a range of dosages (mJ/cm^2^).

The concept of inhibition zone was applied to explore the inhibitory effect of SEOF in a tight channel and was tested on 2-cm- and 1-m-long PTFE channels, which showed a range of UV dosage values (minimum 2.5 to 13.2 mJ/cm^2^) for 6 log reduction of surface-bound *E. coli*, *P. aeruginosa*, and *MRSA*. Previous studies have provided consistent and similar findings regarding the UV dosage required to achieve specific log reductions in surface-bound bacterial populations. Cheng et al. (34), Lanzarini-Lopes et al. (30), and Mohsin et al. (18) reported UV dosages of 11.88, 4.3, and 5.8 mJ/cm^2^ UV dosage, respectively, for a 5-log, 4-log, and 6-log reduction of surface-bound *E. coli* on a nutrient-rich media. Similarly, Mariita et al. (36) reported a UV dosage of 11.76 mJ/cm^2^ for a 4-log reduction of surface-bound MRSA on nutrient-rich media.

However, different inactivation values and UV dosages are reported by other studies that depend on the method of culture growth and experimental conditions. For example, a dosage range of 3 to 20 mJ/cm^2^ and 3.1 to 17 mJ/cm^2^ were reported for a 4-log reduction of planktonic *E. coli* and planktonic *P. aeruginosa* (35, 37–39). In the case of biofilm-bound bacteria, Gora et al. (40) reported a 1.3 ± 0.2 log inactivation for a UV dosage of 8 mJ/cm^2^ for *P. aeruginosa*, which is much lower than surface-bound and planktonic bacterial inactivation at the same dosage. The UV sensitivity of bacteria differs greatly depending on the method of culture growth, i.e., surface-bound, planktonic bacteria in an aqueous suspension and biofilm-bound bacteria. In the context of surface-bound bacteria, the presence of a high cell density in the outer layer of culture droplets poses a challenge for UV radiation to penetrate and effectively reach the inner cells, resulting in limited exposure and subsequent DNA damage (34). A similar mechanism is observed inside biofilms, which provide UV shielding to interior cells that can significantly lower the observed UV sensitivity of organisms (41–44).

In the current set of experiments, even though a 6-log reduction was achieved, sterilization was not achieved in the 1-m PTFE channels using the UV-C SEOFs. In the 1-m channel disinfection experiments, a 6-log reduction of CFU was achieved during same-day.plating due to the inability to detect CFUs. However, when the culture was placed in an overnight incubator at 37°C and then plated, CFUs were detected. Any detection of CFUs indicates that sterilization was not achieved in the 1-m PTFE channel using the UV-C SEOF. Various approaches can be utilized to increase the irradiance of UV-C SEOFs to achieve both sterilization and reduce the time needed for disinfection. These approaches include (i) increasing the scattering coefficient of the fibers (ii) using higher-power UV-C LEDs, (iii) enhancing the coupling efficiency between the UV-C LED and the UV-C SEOF to maximize light transmission into the fiber. Although these approaches will improve the technology, this work demonstrated the potential of UV-C SEOFs to provide high-level disinfection (≥6 log reduction of surface-attached CFUs for medically relevant microorganisms), making them a valuable addition for the disinfection of point-of-use channels and as an added step to the current reprocessing (disinfection) protocol for medical equipment. Furthermore, various non-surgical procedures that benefit from water line disinfection and cleaning do not require sterilization, such as DUWL. These applications still need to comply with national drinking water standards of less than 500 CFU/mL of heterotrophic bacteria (45). Therefore, in these scenarios, using SEOFs for disinfection (~<10 CFU/mL) is a viable option. Additionally, UV-C is known to generate ozone under wavelengths of 240 nm, which can contribute to disinfection. However, in our work, a UV-C wavelength of 265 nm was used, which should not contribute to significant ozone generation (46). Finally, changes to the PTFE during long-term, low irradiance exposure should be analyzed in future studies. Minimum to no damage on PTFE is expected by UV light due to the high stability of the material under UV light and low irradiance available by the SEOF. However, this was not validated in this study.

The survival of a small number of bacterial cells following UV exposure, which did not lead to sterilization, can be attributed to two underlying phenomena: (i) the development of UV resistance by the bacteria and (ii) the failure to reach the minimum irradiance (µW/cm^2^) required for complete inactivation. The first phenomenon was tested by inactivating again in the same manner, i.e., resuspending the surviving CFU in tryptic soy broth (TSB) and irradiating with UV again; this indicated that the bacteria have not developed UV resistance. However, a detailed study is needed to further understand bacterial UV resistance. The second phenomenon was previously reported by Mohsin et al. (18) where they quantified the minimum irradiance needed for complete inactivation of *E. coli* to be 0.38 µW/cm^2^ under continuous UV exposure for a prolonged time (hours) at 265-nm wavelength. In the current study, the bacterial strain inside the 1-m PTFE channel was exposed to a minimum continuous low UV-C irradiance of ~0.23 μW/cm^2^, which could be the reason that few CFU survived the UV exposure. However, a minimum of 0.38 µW/cm^2^ irradiance value was reported for surface-bound *E. coli* strain; a similar study is needed to quantify the minimum irradiance needed for the complete inactivation of surface-bound *P. aeruginosa* and MRSA bacterial strains.

One of the limitations of the study is that the findings presented in this work are specifically for surface-attached microorganisms inside a tight channel and not inhibition of biofilms. Biofilms are structured communities of different microorganisms that attach to a surface and form an extracellular polymeric substance (EPS) layer around them. To make any conclusions related to biofilm inhibition, biofilm formation and analysis essays must be included as part of the study. Future work seeks to better understand the potential for SEOFs for mature and relevant biofilm.

The application of UV-C SEOF in various sectors, including healthcare, water disinfection, water distribution, air purification, and the food industry, holds great promise. These UV-C SEOFs offer an innovative solution to overcome the limitations of traditional UV lamps and LEDs by enabling the disinfection of inaccessible surfaces through the elimination of geometrical restrictions. A previous work has been published on the use of optical fibers for disinfection and biofilm prevention. However, this is the first paper to demonstrate that UV light can be used to disinfect medically relevant organisms present in constricted spaces such as tight channels. These findings can facilitate the design of more efficient reactor systems without any dead zones (non-inactivated areas), ensuring comprehensive disinfection. Light distribution technologies such as UV-C SEOFs will be critical in the widespread implementation of UV technologies for water treatment, maintenance of process equipment, and infection control. Significant work is still needed on both the technology development and application in civil, environmental, and medical spaces.

## MATERIALS AND METHODS

### Bacterial cultivation

Two gram-negative (*E. coli* [ATCC 25922] and *P. aeruginosa* [ATCC 15442]) and one gram-positive (Methicillin-resistant *S. aureus* [ATCC 33592]) were used as test organisms in this study. All strains were stored at −80°C in TSB culture media using sterile 20% glycerol stock solution for *P. aeruginosa* and MRSA and 25% glycerol stock solution for *E. coli*.

Experimental strain cultures were prepared by mixing a 1:10 ratio of the culture stock and TSB. Following overnight (18–24 h) incubation, the culture was once again diluted in a 1:10 ratio with TSB, incubated (37°C), and mixed (140 rpm) until an optical density (OD) of 1 cm^−1^ (10^8^ cells/mL) was achieved. A UV-VIS spectrophotometer was used to measure the OD. Each culture was washed three times by centrifuging (*E. coli* [4,000 rpm, 2 min], *P. aeruginosa* [10,000 rpm, 10 min], and MRSA [4,000 rpm, 5 min]) in a phosphate buffer solution (PBS). During each wash, the supernatant was discarded and the pellet was resuspended in PBS by vortexing. The agar plates were prepared by mixing 10 g tryptic soy agar (TSA) (2291, Sigma-Aldrich) and 27.75 g of Mannitol salt phenol agar (63567, Sigma-Aldrich) in 250 mL of distilled water, separately, then autoclaved for 15 min at 121°C. Approximately, 25 mL of solution was poured into a 10-cm-diameter round polystyrene petri dish and was cooled. TSA agar plates were used for *E. coli* and *P. aeruginosa*, while mannitol salt phenol agar plates were used for MRSA. Agar plates containing bacterial strains were incubated at 37°C for 18 to 24 h. CFUs were counted via the plate streaking technique (47).

### Dose response of bacterial colonies in nutrient-rich media

The inhibitory effect of UV-C SEOF for pathogenic bacteria (*E. coli*) on nutrient-rich media was measured as previously described (30). The UV-C SEOF was set on an agar plate completely inoculated with *E. coli* culture to form a lawn. The UV-C SEOF was placed above the agar as close as possible without touching it through two small holes on the side of the plate. The plate was covered all the time to avoid any contamination. The agar inoculated with *E. coli* culture was irradiated with UV-C for exposure times of 2, 5, and 10 min separately. After UV-C exposure, the plates were incubated at 37°C for 18 to 24 h. The *E. coli* lawn to the fiber on either side was measured to quantify the inhibition zone. Duplicates were performed for each exposure time.

The UV dosage was obtained by multiplying the irradiance at the edge of the lawn by the exposure time. The exact UV irradiance at each distance from the UV-C SEOF was calculated by following a linear source dissipation equation (equation 1). A diffused source of light follows the Inverse-square law (48), where light irradiance (I) decreases as the distance from the source of light increases, as light spreads out over a larger surface. For a linear source of light like the UV-C SEOF, the irradiance is inversely proportional to the distance from the fiber. Therefore, equation (1) was used to model the decrease in irradiance as a function of distance and radius (30), where (*I*_0_) is the light irradiance measured at the source and (*r*_0_) is the radius of the UV-C optical fiber.


equation (1) $$I=\frac{I_{0}.\;r_{0}}{\left(r_{0}\;+\;D\right)\;}$$

### Assessing 2-cm PTFE channel disinfection with high irradiance exposure

The disinfection efficacy of diffuse UV-C radiation for the test microorganisms was first evaluated in partial PTFE channels (2 cm). A 16-cm-long PTFE channel with an internal diameter (ID) of 4.2 mm was autoclaved for sterilization. The channel was capped at one side and filled with 1 mL of TSB bacterial suspension (10^8^ CFU/mL). Subsequently, the second opening in the channel was capped and the full channel was placed in an incubator (37°C) for 30 min on a shaker table (140 rpm) to allow for bacterial attachment. After incubation, both sides of the channel were opened, and the bacterial suspension was drained and washed with 5 mL of sterilized PBS. The channel was cut into eight 2-cm-long pieces to be used as duplicates for the UV-C optical exposure and controls. Two channels were placed adjacent to one another on two UV-C SEOFs, two channels were placed on Cytop-coated control fiber, and two channels were placed on the no-fiber controls. An average irradiance (μW/cm^2^) was measured at the channel locations on each fiber using a spectroradiometer (AvaSpec-ULS2048CL-EVO) with a minimum detection limit of 0.10 μW/cm^2^. The UV-C SEOF average irradiance of 216.25 µW/cm^2^ was ~43 times higher than the control fiber, which averaged 5 µW/cm^2^. The channels were irradiated for exposure times of 2, 5, and 10 min. Subsequently, the channels were suspended in 10 mL TSB separately, vortexed, and a volume of 100-µL serially diluted samples was plated for CFU count. Additionally, the bacterial suspension with channels in TSB was incubated (37°C) overnight for 18 to 24 h and was plated the next day to test for sterilization.

### 1-m PTFE channel disinfection

A 1-m-long and 0.5-mm-diameter UV-C SEOF was used to disinfect the 1-m-long PTFE channel. The material and length of tubing selected in this study serve as a surrogate for channels found in endoscopes, DUWL, and POU water treatment devices. The PTFE channel was first autoclaved for sterilization. The channel was then closed/capped at one side and filled with ~10 mL of TSB bacterial suspension. The second side was then capped, and the entire channel was placed in an incubator (37°C) for 30 min on a shaker table (140 rpm) to allow for bacterial attachment to the inner surface. This preparation method yielded a higher level of consistency (lower standard deviation error) in the CFU count upon replication as compared to a flow-through attachment method. After incubation, the channel was drained and washed with 50 mL of sterilized PBS. The 1-m UV-C SEOF was guided through the channel and was used to irradiate the inside surface area for an exposure time of 16 h. Subsequently, the inner surface area was brushed with an endoscope brush (BR-100-42) from KeySurgical to scrape off the attached organism. The brush tip (2 cm) was cut off and suspended in 10 mL TSB. The suspension was vortexed for 2 min to detach bacterial cells from the brush into the TSB. A volume of 100-µL serially diluted samples was platted for CFU count. Additionally, the bacterial suspension in TSB was incubated (37°C) overnight for 18 to 24 h and was plated the next day to test for sterilization. Duplicates were performed for the irradiated (experimental UV-C SEOF) and control (non-fiber) experiments.

### Statistical analysis

Unpaired *t*-tests were performed to determine the statistical significance of the number of CFUs recovered after UV treatment with UV-C SEOF and the Cytop polymer-coated fibers for exposure times of 2, 5, and 10 min separately. The standard error (SE) and R^2^ of inhibition zone constant (k') in Fig. 2 were calculated using the linear regression function in OriginPro 2021 with intercept fixed at zero. Two-sample group *t*-test was performed to determine the statistical significance between the number of CFUs recovered after UV treatment with UV-C optical fiber compared to the control fiber.
